# Two Types of Non‐Abelian Topological Phase Transitions Under Duality Mapping in 1D Photonic Chains

**DOI:** 10.1002/advs.202511935

**Published:** 2025-10-27

**Authors:** Yufu Liu, Yunlin Li, Jingguang Chen, Xianjun Wang, Haoran Zhang, Fang Guan, Xunya Jiang

**Affiliations:** ^1^ College of Intelligent Robotics and Advanced Manufacturing Fudan University Shanghai 200433 China; ^2^ State Key Laboratory of Surface Physics, Key Laboratory of Micro‐ and Nano‐Photonic Structures (Ministry of Education), and Department of Physics Fudan University Shanghai 200433 China; ^3^ The State Key Laboratory of Surface Physics and the Institute for Nanoelectronic Devices and Quantum Computing Fudan University Shanghai 200433 China

**Keywords:** non‐Abelian topology, orbital degree of freedom, robust edge states

## Abstract

Exploring new topological phases and phenomena is essential in the modern physics. Although non‐Abelian topology with braided nodes in multiple bandgaps systems has been extensively explored, new non‐Abelian phase transitions with nodal line degeneracy still merit further theoretical classifications and experimental validations. Here, photonic chains with coupled *p*‐orbital modes are investigated where two types of non‐Abelian phase transitions are revealed. The first type is the braided‐node type, signified by the Dirac degeneracy node moving into or out of the unit circle. The second type corresponds to the sudden emerging of nodal line degeneracy which intersects with the unit circles. Hidden duality symmetry is unveiled in the rotation parameter space, in which two dual‐systems with identical Abelian topological invariants (specifically, Zak phase and Winding number) can be in distinct non‐Abelian topological phases, separated by the nodal‐line type phase transition. Rich non‐Abelian topological phases can be described by generalized quaternion group *Q*_16_. Robust topological edge states are theoretically predicted by non‐Abelian bulk‐boundary correspondence and further experimentally demonstrated. This work expands new mechanism of non‐Abelian phase transition, paving the way to explore more topological phenomena and topological devices in various wave platforms.

## Graphical abstract

In this work, two types of non‐Abelian phase transitions are revealed. The first type is the braided‐node type, signified by the Dirac degeneracy node moving into or out of the unit circle. The second type corresponds to the emerging of nodal‐line degeneracy which intersects with unit circles. Theoretical analyses and robust edge states, governed by non‐Abelian topological charge, are experimentally verified by 1D photonic chain.

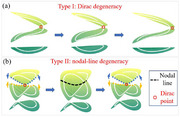

## 1 Introduction

Over the last decade, topological band theory plays an essential role in condense matter physics, fundamentally reshaping our understanding through its discovery of symmetry‐protected topological phases. The tenfold classification,^[^ ^1^, ^2^, ^3^ ^]^ rooted in Altland–Zirnbauer symmetry classes, has successfully unified the description of gapped systems through three fundamental symmetries, that is, time‐reversal symmetry, particle‐hole symmetry, and chiral symmetry. Basically, the tenfold classification only describes single‐gap topology, where the Abelian nature of topological invariants (e.g., Zak phase or Chern number) arises from the commutative of symmetry operations, thus fails to capture multiple bandgap systems. Very recently, it is found that SPTP could go beyond the Abelian classifications.^[^ ^4^, ^5^, ^6^ ^]^ With multiple bandgaps tangled together, the underling new paradigm is characterized by non‐Abelian groups and the corresponding topological invariants are described by non‐Abelian topological charges,^[^ ^7^, ^8^, ^9^ ^]^ such as the quaternion group *Q*_8_. Besides theoretical breakthrough, non‐Abelian topology have also been experimentally realized by photonic,^[^ ^6^, ^10^, ^11^ ^]^ transmission line network^[^ ^8^, ^9^ ^]^ and acoustic^[^ ^12^ ^]^ systems, and relevant phase transitions have also been clearly observed.^[^ ^8^, ^9^, ^13^, ^14^, ^15^ ^]^

**Figure 1:**
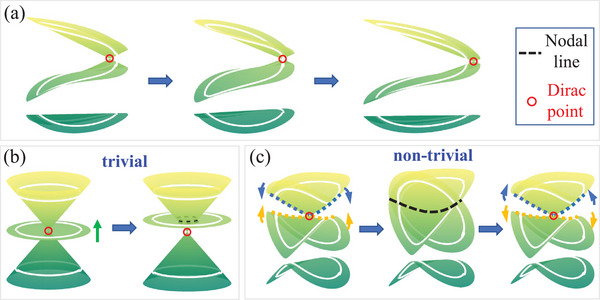
Three configurations of band degeneracy and relevant topological phase transitions.

Generally, non‐Abelian phase transitions and bulk‐boundary correspondence are well investigated through the evolution of band nodes. For instance, the creation, annihilation, splitting and merging of band nodes, can be systematically analyzed through the non‐Abelian topology.^[^ ^11^, ^13^, ^14^, ^15^ ^]^ Similarly, in 1D systems, as schematically depicted in **Figure** **1**a, such phase transitions can be also characterized by the evolving of band nodes either into or out of the unit circle (white lines) in the 2D extended bands.^[^ ^8^, ^9^ ^]^ Besides the braiding of Dirac nodes, new mechanisms of non‐Abelian topological phase transitions still merit further investigation. Nodal lines, the other types of band degeneracy forming closed loops or extended lines in momentum space, have recently extensively explored in 3D topological semimetals, leading to pivotal theoretical classifications^[^ ^16^, ^17^, ^18^, ^19^, ^20^ ^]^ and experimental validations.^[^ ^21^, ^22^, ^23^, ^24^, ^25^, ^26^ ^]^ However, to date, studies on non‐Abelian phase transitions governed by nodal line degeneracy remain scarce both in theoretical predictions and experimental observations, particularly in low‐dimensional (1D or 2D) systems.

Typically, there are two types of phase transitions from Dirac degeneracy to nodal line degeneracy in 2D extended bands. In the first type (Figure 1b), three bands are initially degenerate at a Dirac point. As the middle band moves with change of certain parameter, the middle band crosses the top band, forming a nodal line degeneracy (black dashed line) while preserving the Dirac point. This transition is topologically trivial, as the nodal line is accidental degeneracy and does not intersect the unit circle (white line). Here, we introduce a second type which is topologically nontrivial. As shown in Figure 1c, the top two bands are initially only degenerate at a Dirac point. While the essential parameter of the system changes, these two bands approach each other gradually, until they suddenly reach the *osculation* at the critical value, forming a nodal line (black dashed line) that represents a topological phase transition point. With further change of the essential parameter, two bands will subsequently separate, reforming a Dirac degeneracy again. We note that the phase transition is topologically non‐trivial, which could be evidenced by the nodal line intersecting with the unit circles and signified by changing of non‐Abelian topological charges. Based on the above analysis, realizing topologically non‐trivial nodal line degeneracy in suitable platforms would provide a new paradigm for research on non‐Abelian topological phase transitions.

In this work, we systematically investigate two types of non‐Abelian topological phase transitions in 1D photonic chain by the interactions of *p*‐orbital modes. Hidden duality symmetry is uncovered in the rotation parameter space, in which two dual systems share the same Abelian topological variants (Zak phase and Winding number) but could be in different non‐Abelian topological phases. Rich non‐Abelian topological phases and topological charges, described by generalized quaternion group *Q*_16_, are discovered and verified by the trajectories of eigenstates varying across the first Brillouin zone (FBZ). We demonstrate that topological phase transitions are characterized by two types of band degeneracy in 2D extended bands. The first type is Dirac node degeneracy with Dirac points evolving either in or out of the unit circle. While the second type corresponds to nodal line degeneracy which intersects with the unit circle. At last, the non‐Abelian bulk‐boundary correspondence and the existence of orbital‐induced topological edge states are experimentally verified, which is beyond the Abelian paradigms and robust against structural disorder. Our work lays a foundation for investigating orbital interactions of classical wave systems and the associated non‐Abelian topological phase transition under duality symmetry, offering an experimental platform to engineer multiple bandgaps topology through synthetic orbital hybridization.

## 2 1D Photonic Chains and Tight‐Binding Model

**Figure 2:**
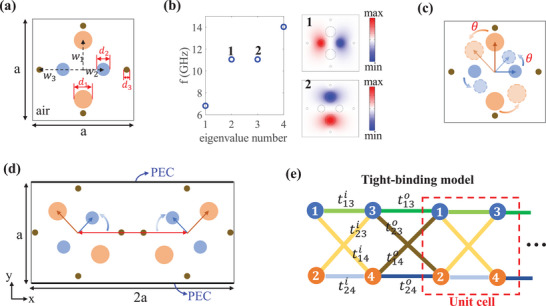
a) The fundamental model of 1D photonic chain. There are three types of rods in the square lattice. b) The eigen‐energies of the fundamental model. Two *p*‐orbital modes are degenerate and the corresponding electric fields are shown in the right panel. c) A new degree of freedom θ is induced to rotate the orientation of two *p*‐orbital modes. d) The unit cell of 1D photonic chain is composed of two fundamental models, where the left one rotates counterclockwise by θ and the right one rotates clockwise by θ. e) The equivalent tight‐binding model.

The fundamental model of 1D photonic chain is shown in **Figure** **2**a, where there are three types of rods in the square lattice, marked by orange, blue and bronze circles, respectively. The orange rods and blue rods are fabricated by different ceramic materials with permittivity ε_1_ = 8.5 and ε_2_ = 5. While bronze rods are fabricated by coppers, which could be approximately regarded as a perfect conductor in microwave band. Here, all materials are considered to be non‐magnetic, with a permeability µ_*r*_ = 1. The diameter and distance of three types of rods are set as *d*_1_ = 0.18*a*, *d*_2_ = 0.12*a*, *d*_3_ = 0.04*a* and *w*_1_ = 0.2*a*, *w*_2_ = 0.16*a*, *w*_3_ = 0.41*a* respectively, where *a* = 0.0225*m* is the width of the square. To further illustrate the *p*‐orbital modes of the model, we calculate the eigen‐energies of the fundamental model, as shown in Figure 2b. Two *p*‐orbital modes are degenerate at *f* = 11GHz, which are localized at two types of rods respectively. In Figure 2c, we introduce a new degree of freedom θ to describe their relative rotation orientation of the two dipoles. We note that the two degenerate *p*‐orbital modes are always orthogonal and do not couple to each other.

Based on the introduction of fundamental model and the rotation regular mentioned above, we further introduce the coupling of *p*‐orbital modes. In Figure 2d, the unit cell of 1D photonic chain is composed of two fundamental models, where the left one rotates counterclockwise by θ and the right one rotates clockwise by θ. In this process, the copper rods are fixed. Therefore, one can easily find that the inversion symmetry is preserved. Crucially, the rotation angle θ controls the hybridization of p‐orbital modes. Physically, rotating the ceramic rods by θ is analogous to tuning pseudospin orientations in quantum systems, which directly modulates the coupling strength between different p‐orbital modes. To further differentiate the intra‐cell and inter‐cell coupling, the distance of left and right fundamental model is set as *D* = 1.13*a*. Meanwhile, in y‐direction, the boundaries are set as perfect electric conductor (PEC) so that the model could be regarded as 1D photonic chain when the unit cell is periodically arranged in x‐direction.

Since two *p*‐orbital modes are both well localized in the rods, there could be approximately regarded as atoms in tight‐binding model,^[^ ^27^ ^]^ as shown in Figure 2e. The equivalent k‐space Hamiltonian can be described as:

(1) $$\begin{array}{c} H\left(k\right)=\left(\begin{array}{c} V\;T\\ T^{†}\;V \end{array}\right),\;T=\left(\begin{array}{c} T_{1}\;T_{2}\\ T_{3}\;T_{4} \end{array}\right) \end{array}$$

where the elements of 2 × 2 matrix *T* and *V* are

(2) $$\begin{array}{cc} & T_{1}=t_{13}^{e}e^{-ik}+t_{13}^{i},\;T_{2}=t_{14}^{e}e^{-ik}+t_{14}^{i},\\ & T_{3}=t_{23}^{e}e^{-ik}+t_{23}^{i},\;T_{4}=t_{24}^{e}e^{-ik}+t_{24}^{i},\\ & V=\left(\begin{array}{c} \omega_{1}\;0\\ 0\;\omega_{2} \end{array}\right) \end{array}$$

where $t_{u(v)}^{i(e)}$ is the coupling strength, the superscript *i* (*e*) represents intra‐cell (extra‐cell) coupling and the subscript *u* (*v*) represents the coupling between u‐atom and v‐atom. ω_1_ and ω_2_ are onsite energy of two *p*‐orbital modes. From Equation (2), it is clear that for θ = 0° (in this case, ω_1_ = ω_2_ and $t_{23}^{i(e)}=t_{14}^{i(e)}=0$), the system can be divided into two subspaces corresponding to two orthogonal *p*‐orbital modes. Each sub‐space corresponds to one copy of the conventional SSH model, and it obeys chiral symmetry. While for θ ≠ 0°, two orthogonal *p*‐orbital modes could couple with each other. The derivations of the Hamiltonian of tight‐binding model are shown in the Section S1 (Supporting Information).

## 3 Results

### 3.1 Hidden Duality Symmetry and Abelian Topological Phases

Duality symmetry is generally regarded as a hidden symmetry that maps unrelated physical systems onto each other,^[^ ^28^, ^29^, ^30^ ^]^ which has been well studied in elastic systems^[^ ^30^ ^]^ and recently discovered in photonic kagome system.^[^ ^31^, ^32^ ^]^ Remarkably, for our 1D photonic chain, the geometric equivalence of clockwise and counterclockwise orbital rotations gives rise to a duality symmetry, in which the the Hamiltonian *H*(*k*) described in Equation (1) satisfies:

(3) $$UH\left(k,t\left(\theta \right)\right)U^{-1}=H\left(k,\theta \right)$$

where $U={\hat{\sigma }}_{0}⊗{\hat{\sigma }}_{z}$ is a unitary operator, $\hat{\sigma_{i}}$ is Pauli matrix, and *t*(θ) is a mapping from the parameter space θ to itself. Here, *t*(θ) satisfies *t*(θ) = π − θ. According to Equation (3), it is clear that the hidden duality symmetry is unveiled, that is, for each sub‐system with rotation θ ∈ (0°, 90°), there is a dual‐system with θ* ∈ (90°, 180°) satisfying θ* = π − θ. Crucially, the critical point with angle θ_*c*_ = 90° is a self‐dual point. The detailed derivations of duality symmetry in 1D photonic chain are presented in Section S2 (Supporting Information).

**Figure 3:**
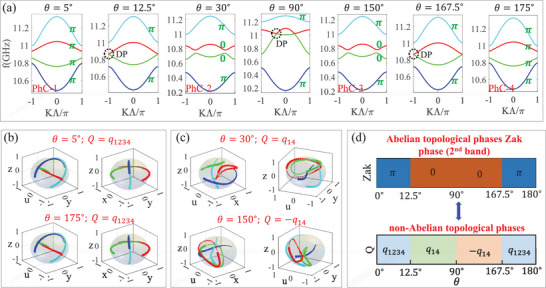
a) The band structures of photonic system with θ varying from 0° to 180°. Four bands are depicted by blue, green, red and cyan lines, respectively. Dirac points (DPs) are marked by black dotted circles and Zak phases are labeled in green letters. PhC‐1, PhC‐2, PhC‐3 and PhC‐4 represent the system with θ = 5°, θ = 30°, θ = 150° and θ = 175°, respectively. b,c) Orthographic projections of the four eigenstate trajectories (shown in different colors) onto 3D solid spheres for θ = 5°, θ = 175°, θ = 30° and θ = 150° respectively. The direction of increasing line width indicates *k* varying from −π to π. Blue, green, red and cyan lines stands for the first, second, third and fourth band. d) The Abelian topological index (Zak phase of 2nd band) and non‐Abelian topological phases in parameter space θ.

In general, two systems related with duality symmetry possess the identical energy spectra.^[^ ^30^ ^]^ To verify this properties, in **Figure** **3**a we depict the bandstructure of the 1D photonic chain with different rotation angle θ. It shows that four energy bands are appeared near *f* = 11*GHz*, which are formed by the coupling of two p‐orbital modes between left and right fundamental model. Specifically, in Figure 3a, PhC‐1 with θ = 5° (PhC‐2 with θ = 30°) and PhC‐4 with θ = 175° (PhC‐3 with θ = 150°) present the same bandstructure, guaranteed by duality symmetry.^[^ ^30^ ^]^ Interestingly, there are two types of band crossing conditions, which usually regarded as the sign of phase transition. For the first type with θ = 12.5° and θ = 167.5°, the second and third band are degenerate at band edge *k*Λ = ±π, forming type‐I Dirac points.^[^ ^33^ ^]^ While for the second type with θ = 90°, the second and third band are crossing at non‐high symmetric point with *k*Λ ≈ ±0.4π, forming type‐II Dirac points.^[^ ^34^, ^35^, ^36^ ^]^

Having established the role of θ in orbital hybridization and the duality symmetry, we the uncover topological phases and phase transition in the 1D photonic chain. We first consider the Abelian index, i.e., Zak phases, of all four bands, which can be calculated by Wilson loop method:^[^ ^37^, ^38^ ^]^

(4) $$\theta_{n}^{Zak}=\sum_{j\in BZ}-Im\left[\ln\left\langle \phi_{n,k_{j}}|\phi_{n,k_{j+1}}\right\rangle \right]$$

where the subscript of $\phi_{n,k_{j}}$ represents the eigenstate of discrete momentum *k*_*j*_ of *n*th band in the FBZ. For tight‐binding model, $\phi_{n,k_{j}}$ can be obtained by solving eigenstates of Equation (1). While for 1D photonic system, $\phi_{n,k_{j}}$ actually represents the eigen‐magnetic field in the unit cell. The Zak phases of PhC‐1, PhC‐2, PhC‐3 and PhC‐4 are labeled in green letters in Figure (3a). For the first type of band crossing condition with θ = 12.5° (θ = 167.5°), it is a clear topological phase transition accompanying by the flip of Zak phases from 0 to π (or from π to 0) for second and third bands. While for the second type with θ = 90°, the Zak phases remain unchanged during the band crossing and re‐opening process, which indicates that it is not a topological phase transition in the Abelian description even with band crossing process. In fact, we can strictly demonstrate that the Abelian topological index (Zak phase and winding number) has to keep unchanged under duality mapping, details are shown in Section S2 (Supporting Information).

### 3.2 Non‐Abelian Topological Phase Transition under Duality Mapping

In the last section, we have shown that θ = 90° is generally not regarded as an Abelian topological phase transition due to that the Abelian invariants remain unchanged, even with band crossing and re‐opening process. However, when we consider multiple bandgaps tangled together, their coupling introduces richer physics that make the classification non‐Alebian, which possess non‐commutative and fruitful braiding structures.^[^ ^7^, ^8^, ^9^ ^]^ Therefore, we will next explore the non‐Abelian classification of our four bands in the 1D photonic chain.

According to Equation (1), the presence of PT symmetry in the four‐band Hamiltonian (that is, *H*(*k*) = H*(*k*)) guarantees that it can be expressed as a real‐valued matrix in an appropriately chosen basis.^[^ ^7^, ^8^, ^9^ ^]^ Thus, the corresponding order‐parameter of Hamiltonian is $M_{4}=O\left(4\right)/Z_{2}^{4}$, where *O*(4) is the 4D orthogonal group and $Z_{2}^{4}$ means that each eigenstate has a gauge freedom of ±1. The fundamental homotopy group of the system can be expressed as π_1_(*M*_4_) = *Q*_16_, where the topological charge could be defined by the eigenstate trajectories along Brillouin zone in k‐space.^[^ ^9^ ^]^

In Figure 3b,c, we calculate the orthographic projections of the four eigenstate trajectories (shown in different colors) onto 3D solid spheres with *k* running across 1D FBZ from −π to π, where the size of the linewidth from small to large indicates the direction of the eigenstate trajectories. Taking θ = 5° (Figure 3b) as an example, it shows that all four eigenstates flip their sign after *k* runs across the 1D FBZ in uyz‐space and uxy‐space, which indicates all four bands are topological non‐trivial. The non‐Abelian charge for PhC‐1 (θ = 5°) can be calculated as *Q* = *q*_1234_. Meanwhile, one can see that rotation directions of four eigenstates of PhC‐4 (θ = 175°) are identical with that of PhC‐1, indicating that they belong to the same non‐Abelian topological phase with charge *Q* = *q*_1234_. Step by step, due to the topological phase transition at θ = 12.5°, for PhC‐2 with θ = 30° in Figure 3c, the non‐Abelian topological charge can be calculated as *Q* = *q*_14_, with the first (blue line) and fourth (cyan line) eigenstates flipping their signs after *k* runs across the 1D FBZ. While for PhC‐3 (the duality system of PhC‐2) with θ = 150° as shown in Figure 3c, the rotation directions of eigenstate trajectories are opposite to those of PhC‐2, indicating that the topological charge of PhC‐3 is *Q* = −*q*_14_. Thus, we show that although PhC‐2 and PhC‐3 possess the identical Abelian index, they could belong to different non‐Abelian phase with non‐Abelian topological charges *Q* = ±*q*_14_ respectively, which are exact two elements in the same conjugacy class. Here, we reveal that the non‐Abelian topological phase transition could occur between two systems related by duality symmetry, even although two sub‐system sharing the same bandstructure and Abelian index (Zak phase and winding number). Furthermore, the non‐Abelian topological phases transition point with θ_*c*_ = 90° is exact the self‐dual point, the non‐Abelian topological charge of which could be ill‐defined.

To summary, we further depict the Abelian and non‐Abelian phase diagrams of the system in parameter space θ, as shown in Figure 3d. For Abelian topological phases, we take the second band as an example and calculate the Zak phase as Abelian index. Comparing the Abelian and non‐Abelian phase diagrams, it shows that the non‐Abelian description expands new topological phases during the duality mapping with *Q* = ±*q*_14_, which are indistinguishable in the Abelian description. Consequently, the new topological phases will induce new topological phenomena, such as bulk‐boundary corresponding that would be carefully discussed in the following section.

### 3.3 Two Types of Non‐Abelian Topological Phase Transitions in 2D Extended Bands

Although the non‐Abelian topological charges are well defined in the 1D system, the phase transition would be more straightforward to visualize if we generalize the 1D Hamiltonian onto a synthetic 2D parameter space. Hence, we further introduce a parameter ρ and make substitutions cos *k* → ρcos *k* = *k*_1_ and sin *k* → ρsin *k* = *k*_2_. Obviously, the original 1D Hamiltonian in *k* space is a unit circle satisfying ρ = 1 (i.e., $k_{1}^{2}+k_{2}^{2}=1$) and the topology charge of original 1D Hamiltonian is characterized by the band degeneracies encircled by unit circle.^[^ ^8^, ^9^ ^]^

**Figure 4:**
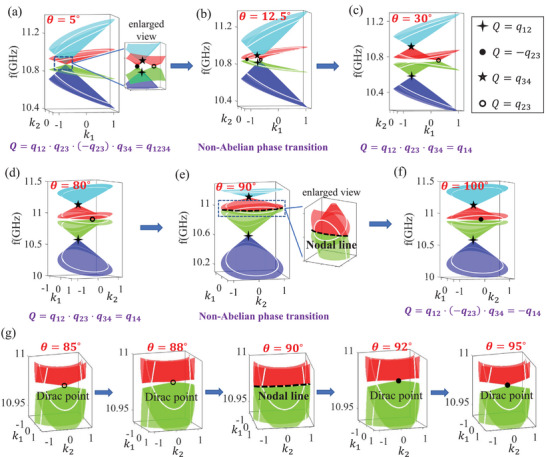
The extended 2D bands in *k*_1_, *k*_2_ space for a) θ = 5°, b) θ = 12.5°, c) θ = 30°, d) θ = 80°, e) θ = 90°, and f) θ = 100°. The topological charges of Dirac points are marked by four‐pointed star, pentagram, solid circle, and hollow circle, respectively. The nodal line in e) is marked by blue dashed line. g) The enlarged view of phase transition near θ = 90°.

First, we investigate the first type of topological phase transition near θ = 12.5°. In **Figure** **4**a, we depict the 2D extended bands for θ = 5° in {*k*_1_, *k*_2_} space, where the white lines represent the original 1D bands. It shows that there are four Dirac nodes inside the unit circle, and the topological charge of nodes could be calculated as *Q* = *q*_12_, *q*_23_, −*q*_23_ and *q*_34_ from bottom band to top band, respectively. Thus, the topological charges of 1D bands could be obtained by the non‐Abelian multiplications of nodes inside the unit circle, that is *Q* = *q*_12_ · *q*_23_ · (− *q*_23_) · *q*_34_ = *q*_1234_, which is consistent with the results by orthographic projections of the four eigenstate trajectories. With the increase of θ, a Dirac point with *Q* = −*q*_23_ (black dot) between 2nd and 3rd bands moves toward the boundary of the unit circle. Critically, as shown in Figure 4b, it intersects with the unit circle when θ = 12.5°, which is generally regarded as a topological phase transition point.^[^ ^8^, ^9^ ^]^ Further increase θ, as shown in Figure 4c, the Dirac point moves out of the unit circle and the topological charge of the 1D band could be calculated as *Q* = *q*_12_ · *q*_23_ · *q*_34_ = *q*_14_, which is also in agreement with the results by orthographic projections of the four eigenstate trajectories. For the other phase transition with θ = 167.5°, the phase transition also accompanies with the Dirac point moving in the unit circle, details are carefully discussed in Section S4 (Supporting Information).

While near θ = 90°, the topological phase transition exhibits distinct characteristics. In Figure 4d, we depict the 2D extended bands for θ = 80° in {*k*_1_, *k*_2_} space, where the topological charge can be calculated as *Q* = *q*_12_ · *q*_23_ · *q*_34_ = *q*_14_. Interestingly, in Figure 4e, for the topological phase transition point with θ = 90°, the second band and third band are degenerate as a nodal line (as shown in the enlarged view). The nodal line cuts the unit circle of two bands, which indicates the topological charge are ill‐defined in this case. Specifically, at the critical point θ = 90°, the first and third bands are completely decoupled from the second and forth bands, inducing discontinuous eigenstates near the dirac point formed between the second and third bands, details are shown in Section S3 (Supporting Information). Furthermore, after the phase transition, the degeneracy of nodal line is lifting as a Dirac node again but with topological charge *Q* = −*q*_23_, as shown in Figure 4f. Hence, the topological charges of 1D bands with θ = 100° is *Q* = *q*_12_ · (− *q*_23_) · *q*_34_ = −*q*_14_. In Figure 4g, we further depict the enlarged view of topological phases transition process near θ = 90°, which clearly exhibits the bands transition from Dirac degeneracy to nodal line degeneracy and then back to Dirac degeneracy during the non‐Abelian topological phase transition. Here, we reveal the second type of topological phase transition with the nodal line degeneracy during phase transition for the first time, which is fundamentally different from the Dirac nodes braiding reported in previous works.^[^ ^8^, ^9^ ^]^ Additionally, to better distinguish these two types of non‐Abelian topological phase transitions, we present a detailed comparison in the Supplementary Material S3 in different aspects, such as the Hamiltonian, physical mechanisms, topological charges and so on.

We note that although previous works^[^ ^8^, ^9^ ^]^ reported type‐I phase transition mediated by Dirac‐node crossing, we uncover a distinct Type‐II non‐Abelian phase transition mechanism governed by nodal‐line degeneracy, through which topological charges reverse (e.g., *q*_14_ → −*q*_14_). Furthermore, by leveraging θ‐modulated orbital hybridization to control topological phase transition, we establish a dynamically configurable platform for on‐demand non‐Ableian phase switching.

### 3.4 Non‐Abelian Bulk‐Boundary Corresponding and Topological Edge States

Bulk‐boundary correspondence, the connection between the bulk topology and the emergence of boundary states, is one of the most fundamental phenomena in condense matter physics. In the Abelian framework, the number of edge states can be described by the difference between two topological invariants across the domain wall. Recently, the bulk‐boundary correspondence has be interpreted in non‐Abelian description by defining a “domain‐wall charge” Δ*Q* = *Q*_*L*_/*Q*_*R*_, where *Q*_*L*_ and *Q*_*R*_ are the topological charges of the left and right samples.^[^ ^9^ ^]^ The domain wall charge Δ*Q* is also an element of the non‐Abelian group and governs the properties of the edge states. Another method to describe the non‐Abelian bulk‐boundary correspondence in 1D system is the 2D extended bands, where the number and location of edge states can be predicted by the Dirac points inside the unit circle of 2D extended bands.^[^ ^8^, ^9^, ^11^ ^]^ In the followings, we will reveal the non‐Abelian bulk‐boundary correspondence in our system during the non‐Abelian topological phase transitions.

**Figure 5:**
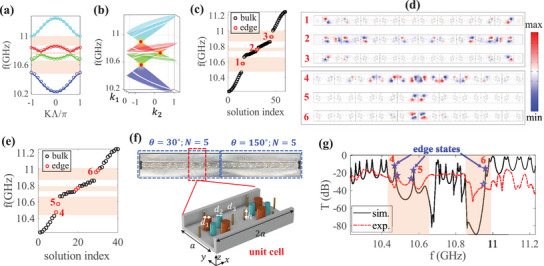
a) The bandstructure of PhC‐2 (lines) and tight‐binding model (circles) with θ = 30°. Red shadows indicate three bandgaps. b) Extended 2D bands with the non‐Abelian topological charges *Q* = *q*_14_. White circles indicate the corresponding 1D bands, and red dots represent DPs. c) The energy spectra of finite PhC‐2 (*N* = 10) with perfect conductor boundary condition, where red circles represent edge states. d) Simulated electric field distributions of edge states. e) The energy spectra of PhC‐2 (*N* = 5) splicing with PhC‐3 (*N* = 5), where red circles represent edge states. f) The experimental structure of 1D photonic chain. Enlarged view: the unit cell of 1D photonic chain. g) The transmission spectra of PhC‐2 (*N* = 5) splicing with PhC‐3 (*N* = 5). The simulated and experimental results are denoted by the black lines and red dotted lines, respectively.

In **Figure** **5**a, we calculate the bandstructure of PhC‐2 with θ = 30° by transfer matrix method (lines) and tight‐binding Hamiltonian (circles), where three complete bandgaps are represented by orange shadows. It shows that the tight‐binding Hamiltonian fits well with the 1D photonic chain. To further discover the non‐Abelian bulk‐boundary correspondence, we then calculate the extended 2D bands in *k*_1_, *k*_2_ space in Figure 5b. Obviously, in each of the three bandgaps, there is a Dirac point within the unit cell (white lines), predicting the emergence of edge states in each bandgap. To demonstrate that, we place finite 1D photonic chain (with *N* = 10) in air and envelop it by a perfect electric conductor (PEC). The energy spectra are calculated in Figure 5c and it exactly shows that there are two degenerate edge states (red circles) in each bandgaps, which is consistent with the theory prediction. The corresponding electric field distributions of edge states are shown in upper panel of Figure 5d, in which the edge states are distinctly dipole‐distributed and well localized at both ends.

To further verify the theoretical values of non‐Abelian topological charges, we splice two topological distinct phases together and show the existence of edge states in the domain‐wall. Thus, we splice PhC‐2 (*Q* = *q*_14_) with *N* = 5 cells in the left side and PhC‐3 (*Q* = −*q*_14_) with *N* = 5 cells in the right side, and the energy spectra are shown in Figure 5e. Due to that the non‐Abelian topological charges are different in each side and the domain‐wall charge can be calculated *Q* = *Q*_*L*_/*Q*_*R*_ = −1, thus there are two edge states (red circles) in each bandgap and the corresponding electric field distributions of edge states are shown in the bottom panel of Figure 5d, in which the edge states are well localized around the domain wall.

Experimentally, the existence of topological edge states could be verified by the transmission peaks inside certain bandgaps from transmission spectra. The experimental 3D structure of 1D photonic chain (with the upper plate removed) is shown in Figure 5f, where we set PhC‐2 with *N* = 5 in the left side and PhC‐3 with *N* = 5 in the right side. The unit cell of PhC‐2 is shown in the enlarged view. Here, the height of rods are set as *h* = 10*mm* so that only fundamental TE10 modes could propagate in the 1D photonic chain. Other structural and material parameters are the same as in Figure 2. The experimental (red dashed line) and simulated (black solid line) transmission spectra are shown in Figure 5g. For the first gap, both simulation and experimental results indicate the presence of two transmission peaks, corresponding to two edge states (marked by “4” and “5”). For the second gap, since the width of second gap is too narrow, so that it is rather difficult to be excited for our finite 1D photonic chain. While for the third gap, both simulation and experimental results show that there is only one transmission peak, marked by “6.” The disappearance of the other edge state due to that it is so proximity to the edge of the fourth band that it could easily merge into the bulk band under the influence of realistic factors such as material absorption and defects. Detailed experimental setups are presented in Section S5 (Supporting Information).

### 3.5 The Roubstness of Topological Edge States

**Figure 6:**
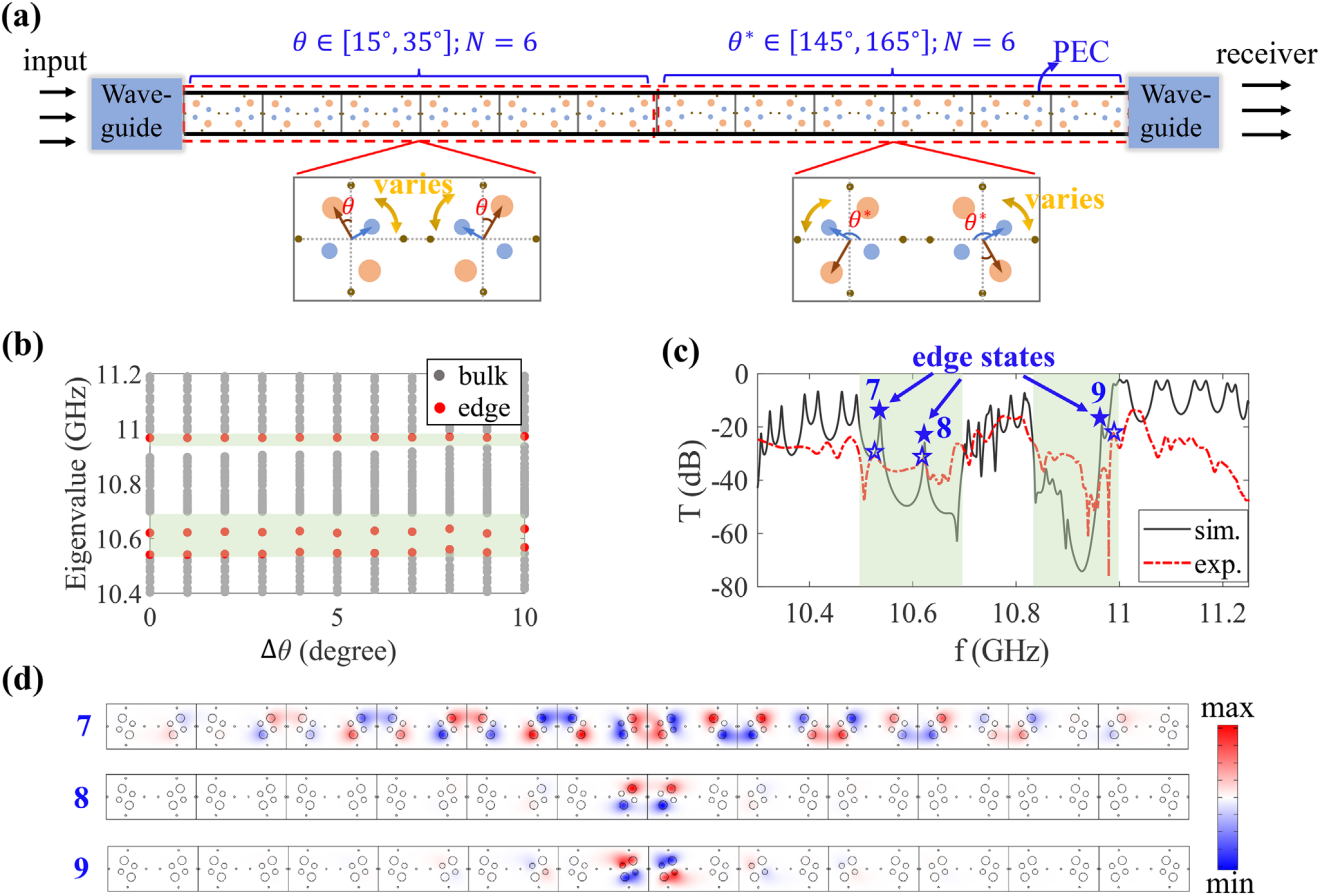
Disorder robustness of topological edge states. a) The illustration of the measurement of the transmission spectra. The left sample and right sample with random rotation angle θ_*L*_ = 25° ± Δθ · γ and θ_*R*_ = 155° ± Δθ · γ, where Δθ is the random strength and γ is an evenly distributed random number in the range [− 1, 1]. b) The average energy spectra with the varying of random strength Δθ, where bulk and edge states are marked by grey and red dots. Here, ten configurations are conducted for each random strength Δθ. c) The transmission spectra of one configuration with random strength Δθ = 5°. The simulated and experimental results are denoted by the black lines and red dotted lines. d) Simulated electric field distributions of edge states.

The robustness of edge states is one of the most exotic properties protected by the bulk topology. We finally investigate the robustness of the non‐Abelian topological edge states against structural disorder. As schematically shown in **Figure** **6**a, we splice two systems in different non‐Abelian topological phases with *Q* = *q*_14_ and *Q* = −*q*_14_ together (as discussed in Figure 5e,g), in which the rotation angle θ of each side is no longer identical but have a random distribution within a certain range. As shown in Figure (5a), for the left side with non‐Abelian topological *Q* = *q*_14_ with *N* = 6 cells, the rotation angle θ is randomly distributed within θ_*L*_ = 25° + Δθ · γ, where Δθ is the random strength, and γ is an evenly distributed random number in the range [− 1, 1]. While for the right side with non‐Abelian topological *Q* = −*q*_14_ with *N* = 6 cells, the rotation angle θ_*R*_ = 155° + Δθ · γ. In Figure (6b), we calculate the energy spectra with the varying of random strength Δθ from [0°, 10°], where bulk and edge states are marked by grey and red dots. Here, ten configurations are conducted for each random strength Δθ and the energy spectra are calculated by the average of these ten configurations. Clearly, two edges states in the first gap and one edge state in the third gap always survive with the increase in random strength Δθ up to 10° because of their topological nature.

To further verify the disorder robustness of topological edge states, we measured the transmission spectra for 1D photonic chain with random strength Δθ = 5°. The experimental (red dashed line) and simulated (black solid line) transmission spectra are shown in Figure 6c. Both simulation and experimental results reveal the robustness of three edge states, marked by ``7 − 9″. And the corresponding electric field distributions of edge states are shown Figure 6d, which are well localized in the domain wall.

## 4 Conclusion

In summary, we have proposed a strategy to utilizing the interactions of *p*‐orbital modes in 1D photonic chain, and demonstrate two types of non‐Abelian topological phase transitions by rotating the orientation of *p*‐orbital modes. We reveal the hidden duality symmetry between different rotation angles θ and θ* = π − θ. Non‐Abelian topological phase transition under duality mapping is unveiled, in which two systems connected with duality symmetry could be in different non‐Abelian topological phases and the self‐dual point is exact the critical topological phase transition point. Physically, the non‐Abelian phase transition can be characterized by two types of band degeneracy in 2D extended bands: the first type is Dirac node degeneracy with Dirac points moving either in or out of the unit circle, while the second type corresponds to nodal line degeneracy which intersects with the unit circle. Finally, bulk boundary correspondence is theoretically discussed and experimentally verified, in which the existences of edge states are robust against structural disorder. Our work relates non‐Abelian topology and hidden duality symmetry in a classical wave system, providing a prospective method for further explorations involving orbital‐dependent devices in various wave platforms. Importantly, future investigations could prioritize two promising avenues: 1) engineering synthetic dimension to research nodal braiding in classical wave systems, and 2) harnessing other DoFs (e.g., polarizations) in photonic systems to investigate novel non‐Abelian topology.

## 5 Experimental Section

### Numerical Simulations

The band structures of 1D photonic chain in Figures 4a and 5a were calculated by transfer matrix method. The energy spectra and electric field distributions in Figure 2b, Figures 5 and 6 were calculated by finite element method using commercial software (COMSOL Multiphysics). The 2D extended bands were calculated by the tight‐binding Hamiltonian in Equation (1).

### Experiments

The designed metal plates were manufactured by the means of metal machining technique and dielectric rods were fabricated by the ceramic materials (geometry tolerance of 0.2mm). The real dielectric rods exhibit intrinsic absorption with permittivity ε_1_ = 8.5 + 0.0017*i* and ε_2_ = 5 + 0.001*i*. In experimental setup, the ports of PNA‐X Network Analyzer N5245B of Keysight are connected to the bonded 1D Photonic chain with coax‐to‐waveguide adapters BJ100 to send and detect the signals. Detailed experimental setup is shown in the Section S5 (Supporting Information).

## Conflict of Interest

The authors declare no conflict of interest.

## Supporting information

Supporting Information

## Data Availability

The data that support the findings of this study are available from the corresponding author upon reasonable request.
