# Supplementary material for: Two Types of Non‐Abelian Topological Phase Transitions Under Duality Mapping in 1D Photonic Chains
Source: Adv Sci (Weinh). 2025 Oct 27;13(2):e11935. doi: 10.1002/advs.202511935 (PMC12786356; doi:10.1002/advs.202511935)
Supplement: Supplementary file 1 — Supporting Information [file ADVS-13-e11935-s001.pdf]

Supplementary Material: Two types of non-Abelian topological phase transitions under duality mapping in one-dimensional photonic chains

Yufu Liu[1, †], Yunlin Li[1, †], Jingguang Chen[2, †], Xianjun Wang[1], Haoran Zhang[1], Fang Guan[3] and Xunya Jiang[1*]

[1]College of Intelligent Robotics and Advanced Manufacturing, Fudan University, Shanghai, 200433, China

[2]State Key Laboratory of Surface Physics, Key Laboratory of Micro- and Nano-Photonic Structures (Ministry of Education), and Department of Physics, Fudan University, Shanghai, 200433, China

[3]The State Key Laboratory of Surface Physics and the Institute for Nanoelectronic Devices and Quantum Computing, Fudan University, Shanghai, China

[*] jiangxunya@fudan.edu.cn

†The three authors contribute equally to this work.

Section 1: The Hamiltonian of 1D photonic chain

Here, we present a detailed derivation of the Hamiltonian of 1D photonic chain. Since the p-orbital modes we considered are well localized in the rods, as shown in Fig. (1b) in the main text, hence, each rod could be approximately regarded as an atom in tight-binding approximation. In Fig. (S1), we illustrate the tight-binding model of 1D Photonic chain. Clearly, there are four atoms in the unit cell, marked by number characters "1~4", where each number represents a p-orbital mode. We note that the on-site energies of four p-orbital modes could be different with the varying of rotation angle θ, which can be calculated by the average energy of split p-orbital modes in energy spectra [1]. The on-site energies of atoms "1" and "3" are set as ω_1, while those of atoms "2" and "4" are ω_2.

Meanwhile, the coupling coefficient could be calculated by the overlapping integral [2]:

$$t_{ij} = \frac{1}{2}\int \left[\left(H_i(x)\right)^* H_j(z) + \left(H_j(x)\right)^* H_i(x)\right] dx \qquad (S1)$$

Where $H_{i(j)}$ represents the magnetic field of i-th (j-th) atom. Clearly, the coupling coefficient between "1" and "2" atoms could be zero, since these two p-orbital modes keep orthogonal with the varying of rotation angle θ. Similarly, the coupling coefficient between "3" and "4" atoms is also zero. Therefore, one can easily obtain that $t_{12} = t_{34} = 0$. Moreover, all coupling configurations of the four atoms are illustrated in the Fig. (S1).

Considering the above discussions of onsite energy and coupling coefficient, the Hamiltonian of tight-binding model in k-space can be written as:

$$H = \begin{pmatrix} \omega_1 & 0 & t_{13}^e e^{-ik} + t_{13}^i & t_{14}^e e^{-ik} + t_{14}^i \\ 0 & \omega_2 & t_{23}^e e^{-ik} + t_{23}^i & t_{24}^e e^{-ik} + t_{24}^i \\ t_{13}^e e^{ik} + t_{13}^i & t_{14}^e e^{ik} + t_{14}^i & \omega_1 & 0 \\ t_{23}^e e^{ik} + t_{23}^i & t_{24}^e e^{ik} + t_{24}^i & 0 & \omega_2 \end{pmatrix} \qquad (S2)$$

Where $t_{u(v)}^{i(e)}$ is the coupling coefficient, the superscript i (e) represents intracellular (extracellular) coupling and the subscript u (v) represents the coupling between u-atom and v-atom. Interesting, it is easy to demonstrate that for two dual systems with θ and

$\theta^* = \pi - \theta$, the signs of t_{14} and t_{23} are reversed (i.e., $t_{14}(\theta) = -t_{14}(\theta^*)$ and $t_{23}(\theta) = -t_{23}(\theta^*)$), while other coupling coefficients remain unchanged. And the critical condition with $\theta = 90°$ satisfies $t_{14} = t_{23} = 0$.

In addition, one can easily find that the chiral symmetry is broken in our system by verifying:

$$\{\hat{H}, \hat{\sigma}_z \otimes \hat{\sigma}_0\} \neq 0 \qquad (S3)$$

Where $\hat{\sigma}_z \otimes \hat{\sigma}_0$ is the chiral symmetry operator and $\hat{\sigma}_i$ is the Pauli operator. This is due to the fact that the onsite energies $\omega_1 \neq \omega_2$.

To conclude this section, we verify the correctness of the Hamiltonian of the tight-binding model. In Fig. (S2), we calculate the band structures of 1D photonic chain (lines) and tight-binding model (circles) with different rotation angle θ. It shows that the tight-binding Hamiltonian fits well with the 1D photonic chain.

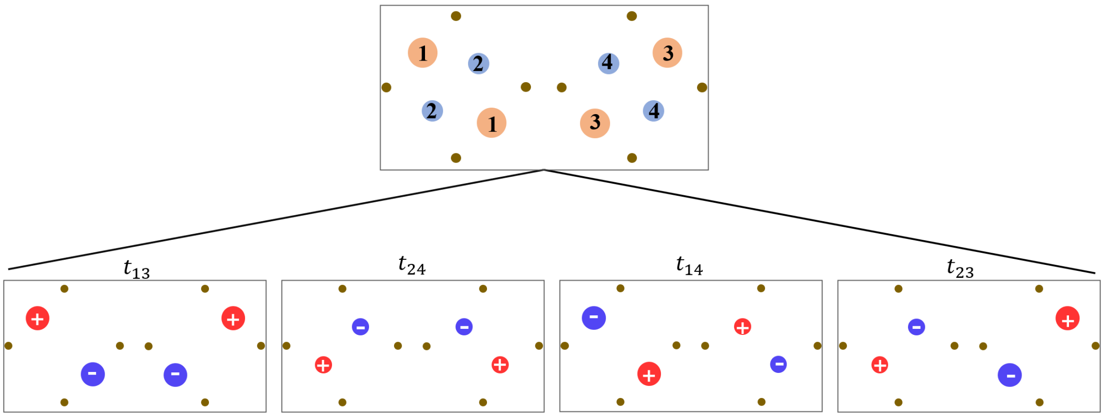

Fig S1. The tight-binding model of 1D photonic chain.

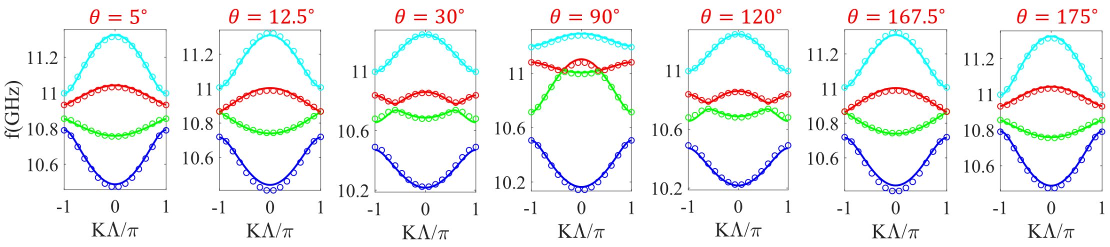

Fig S2. The band structures calculated by 1D photonic chain (lines) and tight-binding model (circles).

Section 2: The Abelian topological invariant under hidden duality symmetry mapping

In this section, we will firstly reveal the hidden duality symmetry of the 1D photonic chain. Then, we will demonstrate that the Abelian topological invariants (including Zak phase and winding number) remain unchanged during the duality symmetry mapping.

In general, symmetries are transformations that commute with the Hamiltonian, rendering the system invariant under such transformations [3]. Duality plays the same role as a special symmetry that maps seemingly unrelated physical systems onto each other via mathematical mapping [4]. As shown in Fig.(S3a), in parameter space P, the sub-space P_1 with Hamiltonian $H(p_1)$ can map to the other sub-space $t(P_1)$ via duality symmetry, where t is a mapping from the parameter space P to itself. Mathematically, it can be expressed as:

$$UH\big(t(p_1)\big)U^{-1} = H(p_1) \qquad (S4)$$

where U is a unitary operator. Specially, when a system maps to itself, then it is called self-dual point, as the red dots shown in Fig.(S3a). In the 1D photonic chain, the parameter space $\Theta \in [0°, 180°]$. Following Eq. (S4), on can easily obtain that the Hamiltonian satisfies:

$$UH\big(k, t(\theta)\big)U^{-1} = H(k, \theta) \qquad (S5)$$

Where $U = \hat{\sigma}_0 \otimes \hat{\sigma}_z$ is a unitary operator, $\hat{\sigma}_i$ is Pauli matrix and $t(\theta)$ is a mapping from the parameter space Θ to itself, satisfying $t(\theta) = \pi - \theta$. One can directly point out that for each sub-system with rotation angle $\theta \in (0°, 90°)$, there is a dual-system with $\theta^* = \pi - \theta \in (90°, 180°)$. Remarkably, the critical point with $\theta_c = 90°$ is a self-dual point that maps the system to itself.

The duality symmetry shows remarkable properties. For instance, dual parts exhibit the identical energy spectra and dynamic characteristics [4]. To illustrate, in Fig.(S3b), we depict the band structure of the system with $\theta = 30°$ and its dual-system with $\theta = 150°$, it clearly shows that these two systems share the same band

structure, which also holds equally for other rotation angle, as shown in the Fig. (2) in the main text.

Except for band properties, the topological research of two systems related with duality symmetry is also an important topic. In the main text, we have shown the non-trivial non-Abelian topological phase transition under the duality mapping. And, in the following, we will illustrate the Abelian properties during the duality mapping.

Considering the Hamiltonian shown in Eq. (S2), we assume that the eigenstate of arbitrary angle $\theta \in (0°, 90°)$ can be expressed as $\phi_\theta = [\phi_1, \phi_2, \phi_3, \phi_4]^T$. Then, the eigenstate of its dual-part with $\theta^* = \pi - \theta$ can be related by Eq. (S5):

$$\phi_{\theta^*} = U^{-1}\phi_\theta = [\phi_1, -\phi_2, \phi_3, -\phi_4]^T \qquad (S6)$$

The above equation indicates that the sign of eigenvalues of second band and fourth band flipped after duality mapping. Then, the Zak phase of each band can be calculated by Wilson loop method [5, 6]:

$$\theta_n^{Zak} = \sum_{j \in BZ} -Im\left[\ln\left\langle \phi_{n,k_j} \middle| \phi_{n,k_{j+1}} \right\rangle\right] \qquad (S7)$$

where the subscript of ϕ_{n,k_j} represents the eigenstate of discrete momentum k_j of n-th band in the first Brillouin zone (FBZ). After calculating the Eq. (S7), it is clear that two dual-systems contribute to the same Zak phase for each band, due to those two negative signs cancel each other out in inner product process. In Fig. (S3b), the Zak phases of each band are labeled in green letters. It shows that two dual-systems are in the same Abelian topological phases with identical Zak phases for four bands.

Besides the Zak phase, we can also easily verify that two dual-systems share same winding numbers. Generally speaking, the chiral symmetry of 1D photonic chain is broken since $\omega_1 \neq \omega_2$. To simplify the discussion of the winding number of the system, we assume that $\omega_1 = \omega_2 = 0$, so that the winding number is quantized. In this case, the Hamiltonian can be rewritten as:

$$H = \begin{pmatrix} 0 & T \\ T^\dagger & 0 \end{pmatrix}; T = \begin{pmatrix} t_{13}^e e^{-ik} + t_{13}^i & t_{14}^e e^{-ik} + t_{14}^i \\ t_{23}^e e^{-ik} + t_{23}^i & t_{24}^e e^{-ik} + t_{24}^i \end{pmatrix} \qquad (S8)$$

Then, the winding number of the Hamiltonian is defined as [1, 7]:

$$W = \frac{i}{2\pi}\int_{-\pi}^{\pi} dk \frac{d \ln \det T(k)}{dk} = -\frac{1}{2\pi}\int_{C} d \arg \det T(k) \qquad (S9)$$

Where C represents a contour swept by $T(k)$ with k varying across the FBZ. As we have discussed above, for a system $\theta \in (0,90^\circ)$ and its dual-system with $\theta^* = \pi - \theta$, one can find that the off-diagonal item of matrix T satisfies $t_{14}(\theta) = -t_{14}(\theta^*)$ and $t_{23}(\theta) = -t_{23}(\theta^*)$. Therefore, the $\det T(k)$ of two dual-systems are identical, which indicates that two systems related with duality symmetry possess the same winding number.

Finally, we can further depict the Abelian and non-Abelian phase diagrams of the system in parameter space θ. For Abelian topological phases, we take the second band as an example and calculate the Zak phase as Abelian index. For Abelian description, we conclude that the Abelian invariant remain unchanged under the duality symmetry mapping. However, the non-Abelian description expands new topological phases during the duality mapping, which could well explain the appearance of topological edge states, as discussed in the main text.

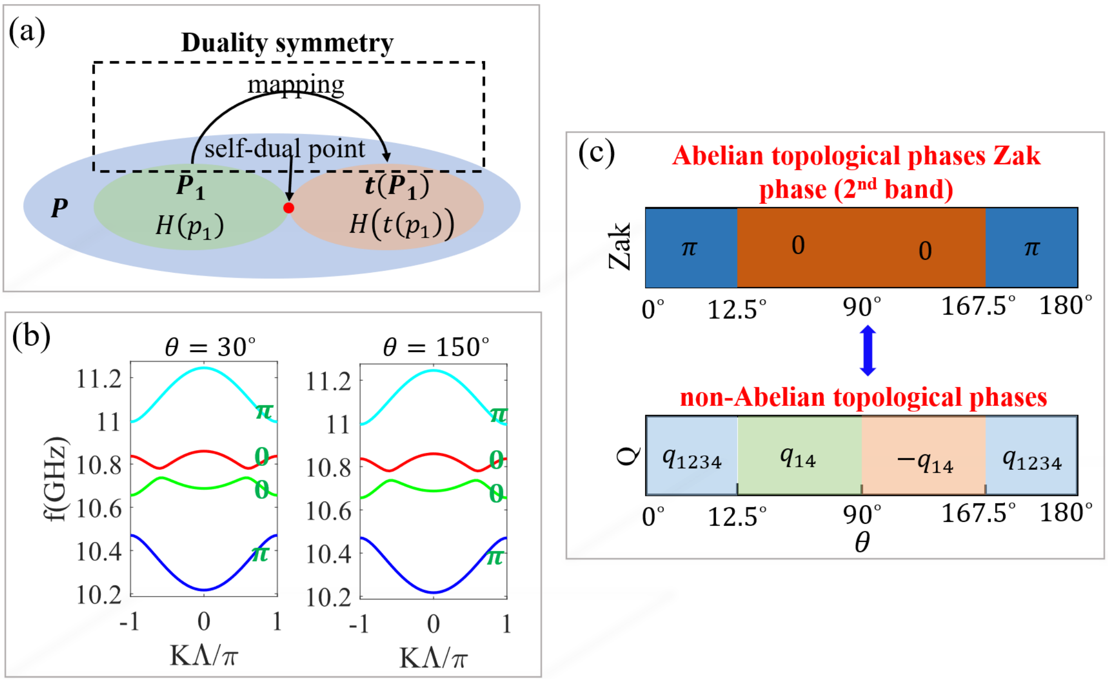

Fig S3. (a) The illustration of mathematical mapping of duality symmetry. (b) The band structures of 1D photonic chain with $\theta = 30^\circ$ and its dual part with $\theta = 150^\circ$, where Zak phases are labeled in green letters. (c) The Abelian topological index (Zak phase of 2nd band) and non-Abelian topological phases in parameter space θ.

Section 3: Ill-defined Non-Abelian charge and topological phase transition via nodal-line degeneracy

In the main text, we observe two distinct types of non-Abelian topological phase transitions. The first corresponds to Dirac point transitions with Dirac points moving in or out of the unit circle. The second involves nodal-line degeneracies, at which the non-Abelian topological charge is ill-defined. In this section, we first present the derivation of non-Abelian charges in our four-band system. Subsequently, we provide a detailed analysis of topological phase transitions via nodal-line degeneracies.

- **Calculating non-Abelian topological charges in four band models**

In this work, we follow Ref.9 to calculate the non-Abelian topological charges in our four band models. The general Wilson operator can be written as:

$$W = exp\left[\oint A(k) \cdot dk\right] \tag{S10}$$

where $[A(k)]_n^m = \langle u_k^m | \partial_k u_k^n \rangle$ is $\mathcal{SO}(4)$-value 1-form Berry connection. First, we decompose the Berry connection into $\mathcal{SO}(4)$ basis:

$$[A(k)] = \sum_{i<j} \beta^{i,j}(k)\, L_{i,j} \tag{S11}$$

where $\mathcal{SO}(4)$ basis can be written as:

$$L_{12} = \begin{pmatrix} 0 & -1 & 0 & 0 \\ 1 & 0 & 0 & 0 \\ 0 & 0 & 0 & 0 \\ 0 & 0 & 0 & 0 \end{pmatrix};\ L_{13} = \begin{pmatrix} 0 & 0 & -1 & 0 \\ 0 & 0 & 0 & 0 \\ 1 & 0 & 0 & 0 \\ 0 & 0 & 0 & 0 \end{pmatrix};\ L_{14} = \begin{pmatrix} 0 & 0 & 0 & -1 \\ 0 & 0 & 0 & 0 \\ 0 & 0 & 0 & 0 \\ 1 & 0 & 0 & 0 \end{pmatrix};$$

$$L_{23} = \begin{pmatrix} 0 & 0 & 0 & 0 \\ 0 & 0 & -1 & 0 \\ 0 & 1 & 0 & 0 \\ 0 & 0 & 0 & 0 \end{pmatrix}; L_{24} = \begin{pmatrix} 0 & 0 & 0 & 0 \\ 0 & 0 & 0 & -1 \\ 0 & 0 & 0 & 0 \\ 0 & 1 & 0 & 0 \end{pmatrix}; L_{14} = \begin{pmatrix} 0 & 0 & 0 & 0 \\ 0 & 0 & 0 & 0 \\ 0 & 0 & 0 & -1 \\ 0 & 0 & 1 & 0 \end{pmatrix} \tag{S12}$$

Then, we lift the Berry connection from Lie algebra $\mathcal{SO}(4)$ into $\mathcal{Spin}(4)$ basis as:

$$[\bar{A}(k)] = \sum_{i<j} \beta^{i,j} t_{i,j} \tag{S13}$$

where $t_{i,j} = -\frac{1}{4}[\Gamma_i, \Gamma_j]$ and Γ_i satisfying:

$$\Gamma_1 = -\sigma_2 \otimes \sigma_1; \Gamma_2 = -\sigma_2 \otimes \sigma_2; \Gamma_3 = -\sigma_2 \otimes \sigma_3; \Gamma_4 = \sigma_1 \otimes \sigma_0 \tag{S14}$$

Where σ_i is i −th Pauli matrix. Then, the generalized quaternion Q_{16} acquired along

a closed loop can be expressed as:

$$Q = exp\left[\oint \bar{A}(k) \cdot dk\right] \qquad (S15)$$

Finally, we calculate the integral of Eq.(S15) and obtain the non-Abelian charge. The quaternion group elements Q are represented by the following mapping:

$$e_1 \to q_{12},\ e_2 \to q_{13}, e_3 \to q_{14},\ e_{12} \to q_{23}, e_{13} \to q_{24},\ e_{23} \to q_{34}, e_{123} \to q_{1234} \quad (S16)$$

where e_i is the basis of Clifford algebra $Cl_{0,3}$:

$$e_1 = \begin{pmatrix} -i & 0 & 0 & 0 \\ 0 & i & 0 & 0 \\ 0 & 0 & -i & 0 \\ 0 & 0 & 0 & i \end{pmatrix};\ e_2 = \begin{pmatrix} 0 & 1 & 0 & 0 \\ -1 & 0 & 0 & 0 \\ 0 & 0 & 0 & 1 \\ 0 & 0 & -1 & 0 \end{pmatrix};\ e_3 = \begin{pmatrix} 0 & -i & 0 & 0 \\ -i & 0 & 0 & 0 \\ 0 & 0 & 0 & i \\ 0 & 0 & i & 0 \end{pmatrix}$$

$$e_{12} = e_1 e_2 = \begin{pmatrix} 0 & -i & 0 & 0 \\ -i & 0 & 0 & 0 \\ 0 & 0 & 0 & -i \\ 0 & 0 & -i & 0 \end{pmatrix};\ e_{13} = e_1 e_3 = \begin{pmatrix} 0 & -1 & 0 & 0 \\ 1 & 0 & 0 & 0 \\ 0 & 0 & 0 & 1 \\ 0 & 0 & -1 & 0 \end{pmatrix}$$

$$e_{23} = e_2 e_3 = \begin{pmatrix} -i & 0 & 0 & 0 \\ 0 & i & 0 & 0 \\ 0 & 0 & i & 0 \\ 0 & 0 & 0 & -i \end{pmatrix}; e_{123} = e_1 e_2 e_3 = \begin{pmatrix} -1 & 0 & 0 & 0 \\ 0 & -1 & 0 & 0 \\ 0 & 0 & 1 & 0 \\ 0 & 0 & 0 & 1 \end{pmatrix} \qquad (S17)$$

Topologically, the non-Abelian charge Q of our system can be also labelled with band-index, which straightforward relates to the Zak phase of each band physically [9]. For example, the non-Abelian topological charge $Q = q_{14}$ indicates that both first band and fourth band acquire Zak phase of π via the rotation of the eigenstates. Specifically, the Hamiltonian H(k) in Eq.(S2) can be written as:

$$H(k) = R(k) I(k) R(k)^T \qquad (S18)$$

where $R(k) \in SO(4)$ is the four-dimensional orthogonal group, $k \in [-\pi, \pi]$ is in the FBZ and $I = diag(\omega_{10}, \omega_{20}, \omega_{30}, \omega_{40})$ is the eigenvalues of $H(k)$. When k runs across the FBZ ($k = -\pi \to \pi$), rotation matrix $R(k)$ continuously acts on eigenstates, and one finally obtains identical (flipped) eigenstates corresponding to a Zak phase of 0 (π). Thus, the non-Abelian topological charge could be directly defined by the eigenstate trajectories and rotation directions along a closed path (1st FBZ).

- **Non-Abelian topological phase transition via nodal line degeneracy**

For the convenience of following discussions, the Hamiltonian described in Eq.(S2) can be rewritten as:

$$H = \begin{pmatrix} \omega_1 & 0 & \Delta t_{13}(k) & \Delta t_{14}(k) \\ 0 & \omega_2 & \Delta t_{23}(k) & \Delta t_{24}(k) \\ \Delta t_{13}^*(k) & \Delta t_{14}^*(k) & \omega_1 & 0 \\ \Delta t_{23}^*(k) & \Delta t_{24}^*(k) & 0 & \omega_2 \end{pmatrix} \qquad (S19)$$

where $\Delta t_{13}(k) = t_{13}^e e^{-ik} + t_{13}^i$, $\Delta t_{14}(k) = t_{14}^e e^{-ik} + t_{14}^i$, $\Delta t_{23}(k) = t_{23}^e e^{-ik} + t_{23}^i$ and $t_{24}(k) = t_{24}^e e^{-ik} + t_{24}^i$. The eigenstate of Eq.(S19) is assumed as $\phi = [u_1; u_2; u_3; u_4]$. By unitary transform of Eq.(S19), the Hamiltonian can be rewritten as:

$$\widetilde{H} = U^+HU = \begin{pmatrix} \omega_1 & \Delta t_{13}(k) & 0 & \Delta t_{14}(k) \\ \Delta t_{13}^*(k) & \omega_1 & \Delta t_{23}^*(k) & 0 \\ 0 & \Delta t_{23}(k) & \omega_2 & \Delta t_{14}(k) \\ \Delta t_{14}^*(k) & 0 & \Delta t_{14}^*(k) & \omega_2 \end{pmatrix} \qquad (S20)$$

where U is a unitary matrix:

$$U = \begin{pmatrix} 1 & 0 & 0 & 0 \\ 0 & 0 & 1 & 0 \\ 0 & 1 & 0 & 0 \\ 0 & 0 & 0 & 1 \end{pmatrix} \qquad (S21)$$

Then, the eigenstates can be described as $\widetilde{\phi} = U^+\phi = [u_1; u_3; u_2; u_4]$.

Next, we focus on the second type of topological phase transition with nodal line degeneracy near $\theta_0 = 90°$. Considering a complete topological phase transition process where θ continuously varies from $\theta_1 = \theta_0 - \Delta\theta$ to θ_0 and then to $\theta_2 = \theta_0 + \Delta\theta$, with $\Delta\theta << \theta_0$. According to the integral overlapping calculated by Eq.(S1), one can obtain that the coupling coefficients Δt_{14} and Δt_{23} change their signs during the topological phase transition process, specifically, $\Delta t_{14}(\theta_0 - \Delta\theta) = -\Delta t_{14}(\theta_0 + \Delta\theta)$ and $\Delta t_{23}(\theta_0 - \Delta\theta) = -\Delta t_{23}(\theta_0 + \Delta\theta)$. Crucially, at the topological phase transition (nodal line degeneracy) with $\theta_0 = 90°$, it satisfies $\Delta t_{23} = \Delta t_{14} = 0$. Under this condition, the Hamiltonian in Eq.(S20) can be diagonalized as:

$$\widetilde{H}_0 = \begin{pmatrix} \omega_1 & \Delta t_{13}(k) & 0 & 0 \\ \Delta t_{13}^*(k) & \omega_1 & 0 & 0 \\ 0 & 0 & \omega_2 & \Delta t_{14}(k) \\ 0 & 0 & \Delta t_{14}^*(k) & \omega_2 \end{pmatrix} = \begin{pmatrix} \widetilde{H}_1(k) & 0 \\ 0 & \widetilde{H}_2(k) \end{pmatrix} \qquad (S22)$$

where $\widetilde{H}_1(k)$ and $\widetilde{H}_2(k)$ are 2×2 matrices. Eq.(S22) indicates that subsystem {u_1, u_3} is completely decoupled from the other subsystem {u_2, u_4}.

Then, we discuss the topology of the critical system at $\theta_0 = 90°$. When considering the one-dimensional k-space at $\theta_0 = 90°$, the second and third bands form a Dirac point through degeneracy (as shown in Fig. 3a of the main text). Notably, the

complete decoupling between subsystem {u1, u3} and subsystem {u2, u4} induces discontinuity in the wave functions of the second and third bands near the Dirac point. Consequently, the trajectories of eigenstates become discontinuous in 4D space, precluding an ill-defined non-Abelian topological charge**.**

When θ derivates from θ_0 (e.g., $\theta_1 = \theta_0 - \Delta\theta$ and $\theta_2 = \theta_0 - \Delta\theta$), the system exhibits non-Abelian properties because all four sites are coupling with each other. Further, given the sign reversal properties of Δt_{14} and Δt_{23} at θ_1 and θ_2 (e.g., $\Delta t_{14}(\theta_0 - \Delta\theta) = -\Delta t_{14}(\theta_0 + \Delta\theta)$ and $\Delta t_{23}(\theta_0 - \Delta\theta) = -\Delta t_{23}(\theta_0 + \Delta\theta)$), the eigenstates at θ_1 and θ_2 satisfy:

$$\tilde{\phi}(\theta_1) = [u_1; u_3; u_2; u_4], \tilde{\phi}(\theta_2) = [u_1; u_3; -u_2; -u_4] \qquad (S23)$$

Consequently, the Berry connection for systems at θ_1 and θ_2 can be derived as:

$$A(\theta_1) = \begin{pmatrix} \langle u_1|\partial_k u_1\rangle & \langle u_1|\partial_k u_2\rangle & \langle u_1|\partial_k u_3\rangle & \langle u_1|\partial_k u_4\rangle \\ \langle u_2|\partial_k u_1\rangle & \langle u_2|\partial_k u_2\rangle & \langle u_2|\partial_k u_3\rangle & \langle u_2|\partial_k u_4\rangle \\ \langle u_3|\partial_k u_1\rangle & \langle u_3|\partial_k u_2\rangle & \langle u_3|\partial_k u_3\rangle & \langle u_3|\partial_k u_4\rangle \\ \langle u_4|\partial_k u_1\rangle & \langle u_4|\partial_k u_2\rangle & \langle u_4|\partial_k u_3\rangle & \langle u_4|\partial_k u_4\rangle \end{pmatrix};$$

$$A(\theta_2) = \begin{pmatrix} \langle u_1|\partial_k u_1\rangle & \langle u_1|\partial_k u_2\rangle & -\langle u_1|\partial_k u_3\rangle & -\langle u_1|\partial_k u_4\rangle \\ \langle u_2|\partial_k u_1\rangle & \langle u_2|\partial_k u_2\rangle & -\langle u_2|\partial_k u_3\rangle & -\langle u_2|\partial_k u_4\rangle \\ -\langle u_3|\partial_k u_1\rangle & -\langle u_3|\partial_k u_2\rangle & \langle u_3|\partial_k u_3\rangle & \langle u_3|\partial_k u_4\rangle \\ -\langle u_4|\partial_k u_1\rangle & -\langle u_4|\partial_k u_2\rangle & \langle u_4|\partial_k u_3\rangle & \langle u_4|\partial_k u_4\rangle \end{pmatrix} \qquad (S24)$$

The off-diagonal components of the Berry connections in these two systems exhibit opposite signs, resulting in distinct non-Abelian topological charges. Specifically, Type-II transition (nodal line degeneracy) occurs between different elements within the same conjugacy class, which manifests opposite orientations of eigenstates trajectory in 4D parameter space. To demonstrate this, we depict the energy spectra of two sub-systems before and after the topological phase transition in Fig. (S4a). It shows that two sub-systems possess identical energy spectra under perfect conductor boundary condition, confirming that they share the same conjugacy class. However, Fig. (S4b) reveals unambiguous emergence of edge states in the first and third gaps when these subsystems are spliced, which further indicates that their topological charges constitute different elements within the same conjugacy class, which is also regarded as a special case of non-Abelian topological phase transition [8,9].

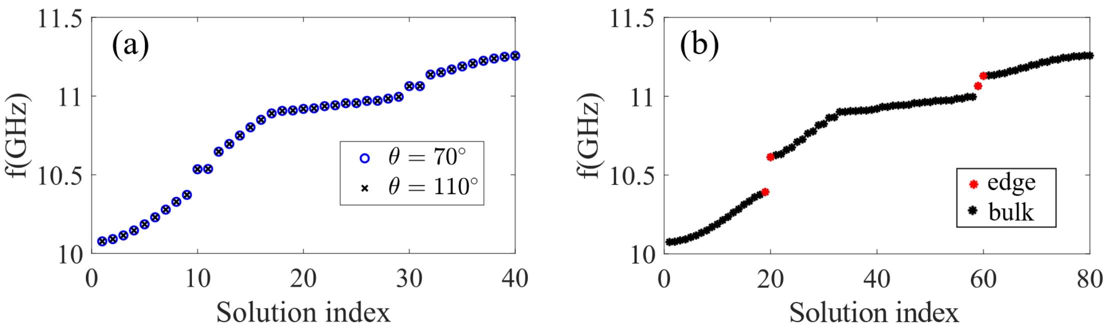

Fig S4. (a) The energy spectra of finite PhC with $\theta = 70°$ (blue circles) and $\theta = 110°$ (black crosses) with perfect conductor boundary condition. (b) The energy spectra of PhC with $\theta = 70°$splicing with PhC with $\theta = 110°$, where red stars represent edge states.

In summary, we conclude the fundamental distinctions between the two types of topological phase transitions. Firstly, Type-I transition manifests the evolving of Dirac points into (or out of) the unit circle within the 2D parameter space, consequently constraining degeneracies in 1D k-space at high-symmetry points ($ka = \pm\pi$), whereas Type-II transition exhibits nodal line degeneracies in the 2D parameter space, permitting degeneracies at arbitrary k in 1D k-space. Topologically, Type-I transition happens between distinct non-Abelian topological charges, while Type-II transition occurs between different elements within the same conjugacy class. Structurally, the Hamiltonians of Type-I transition is non-block-diagonalizable, in contrast to the Type-II transition which is automatically block-diagonalized. Finally, Type-II transition occurs at self-dual points and maps two sub-systems with duality symmetry.

Table S1. The comparison between two types of non-Abelian topological phases transition.

Comparison	**Type-I phase transition**	**Type-II phase transition**
**2D parameter space**	Dirac point moving in/out of the unit circle	Nodal line degeneracy
**1D k-space**	Dirac degeneracy at high-symmetry point $ka = \pm\pi$	Dirac degeneracy at arbitrary k
**Topological phase transition**	Transition between arbitrary distinct non-Abelian charges	Transition between two elements in the same conjugacy class
**Hamiltonian**	Non-block-diagonalizable	Automatically block-diagonalized
**Duality symmetry**	Not applicable	Occurs at self-dual point

Section 4: The verification of nodal line degeneracy by Finite Element Method simulation

In the main text, we discover a new type of non-Abelian topological phase transition characterized by nodal line degeneracy. The nodal line degeneracy manifests in 2D parameter space $\{k_x, k_y\}$ (or $\{\rho, k\}$), projecting onto two Dirac points in conventional 1D band structure in k-space, which is indistinguishable from the first type of phase transition with Dirac degeneracy according to the conventional band measurements. Thus, it is rather difficult to observe and experimentally measure nodal line degeneracy in real systems.

To overcome this limitation, we design a tunable 1D photonic chain and employ Finite Element Method (FEM) simulations to further verify the nodal line degeneracy. Mapping the Hamiltonian into 2D parameter space $\{\rho, k\}$ yields:

$$H(\rho,k) = \begin{pmatrix} \omega_1 & 0 & t_{13}^e \cdot \rho e^{-ik} + t_{13}^i & t_{14}^e \cdot \rho e^{-ik} + t_{14}^i \\ 0 & \omega_2 & t_{23}^e \cdot \rho e^{-ik} + t_{23}^i & t_{24}^e \cdot \rho e^{-ik} + t_{24}^i \\ t_{13}^e \cdot \rho e^{-ik} + t_{13}^i & t_{14}^e \cdot \rho e^{-ik} + t_{14}^i & \omega_1 & 0 \\ t_{23}^e \cdot \rho e^{-ik} + t_{23}^i & t_{24}^e \cdot \rho e^{-ik} + t_{24}^i & 0 & \omega_2 \end{pmatrix} \quad (S25)$$

where ρ is an artificial polar radius where $\rho = 1$ corresponds to the physical 1D photonic chain. Crucially, in Eq. (S25), it shows that modulating the extra-cell coupling $t_{i,j}^e$ emulates variation in polar radius ρ.

We then implement this control through tunable side copper rods (in Fig. (S5a)). The distance of the side copper rods to the center is tunable and set as $w_3 = w_{30} + \Delta w$, where $w_{30} = 0.41a$ corresponding to $\rho = 1$ is defined in the main text. By modulating w_3, we can directly modulate extra-cell coupling $t_{i,j}^e$ and further control the polar radius ρ. In Fig. (S5b), we calculate the evolution of ρ versus w_3 by FEM simulations and overlapping integral in Eq. (S1). It reveals a near-linear decrease in ρ versus w_3 within $w_3 \in [0.96w_{30}, 1.008w_{30}]$, with $w_3 = w_{30}$ corresponding to $\rho = 1$.

The nodal-line degeneracy is unambiguously demonstrated in Fig. (S5c). FEM-computed bands (2nd/3rd) in the reconstructed 2D parameter space $\{k_x, k_y\} = \{\rho \cos k, \rho \sin k\}$ exhibit nodal line degeneracy. This nodal line (red dashed line) shows excellent agreement with tight-binding model (TBM) predictions (blue solid line), verifying the theoretical framework.

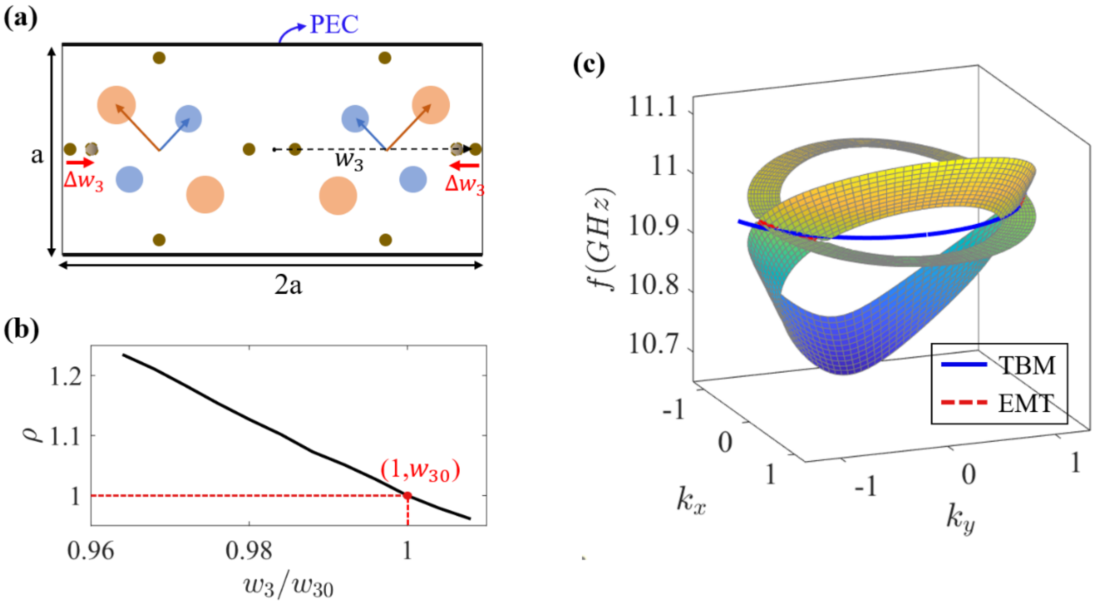

Fig S5. Tunability and nodal line validation in 1D photonic chain. (a) Schematic of side copper rods position adjustment. The distance of side copper rods to the center is tuned as is $w_3 = w_{30} + \Delta w$, where $w_{30} = 0.41a$ (defined in the main text). (b) Evolution of coupling ratio ρ versus w_3. (c) The band structures (second band and third band) in 2D parameter space $\{k_x, k_y\}$ calculated by FEM simulation, where the nodal line calculated by EMT (red dashed line) is well consistent with the TBM results (blue solid line).

Section 5: The edge states and bulk-boundary correspondence of non-Abelian topological charge $Q = q_{1234}$

In the main text, we have detailly demonstrated the bulk-boundary correspondence of 1D photonic chain with non-Abelian topological charge $Q = \pm q_{14}$. In this section, we will introduce the edge states and bulk-boundary correspondence of the other non-Abelian topological phase, i.e., with non-Abelian topological charge $Q = q_{1234}$.

In Fig. (S3c), we show that the non-Abelian topological charge is $Q = q_{1234}$ when $\theta \in (0,12.5°)$. Here, we set $\theta = 5°$ and discuss the bulk-boundary correspondence. In Fig. (S6a), we calculate the band structures of 1D photonic chain (lines) and its corresponding tight-binding approximation (circles), which fits well with each other. As we have mentioned in the main text, one method to describe the non-Abelian bulk-boundary correspondence is expanding 1D bands into 2D extended bands. Hence, we make substitutions such as $\cos k \rightarrow \rho \cos k = k_1$ and $\sin k \rightarrow \rho \sin k = k_2$, and then calculate the extended 2D bands in $\{k_1, k_2\}$ space in Fig. (S6b), where white circle lines indicate the corresponding 1D bands with $\rho = 1$. In the enlarged view, it clearly shows that there are two Dirac points in the midgap within white circle lines, which indicates there are totally four edge states in the midgap according to bulk-boundary correspondence. To verify this, we calculate the energy spectra and filed distributions of 1D finite tight-binding model with $N = 15$ cells under open boundary condition in Fig. (S6c). Four topological edge states, marked by red dots, are degenerate pairwise and locate in the midgap. And the field distributions of edge states show that there are well localized in the boundary.

The similar phenomena are also applied in 1D photonic chain. As illustrated in Fig. (S6d), the 1D photonic chain with 10 cells is enclosed by perfect electric conductor. The energy spectra of 1D photonic chain are shown in Fig. (S6e), where four topological edge states, marked by red circles, locate in the midgap, which is similar to the results of tight-binding model. The corresponding electric field

distributions of edge states are shown in Fig. (S6f), which are well localized in the boundary.

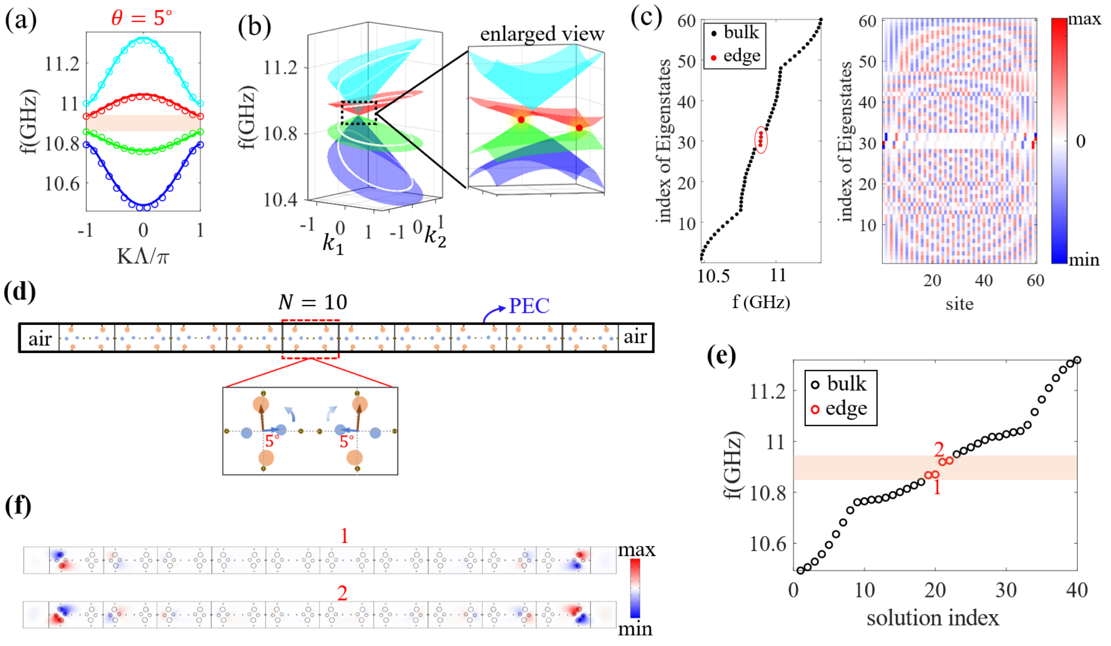

Fig S6. (a) The band structures of 1D photonic chain with $\theta = 5°$ (lines) and tight-binding model (circles). Red shadows indicate three bandgaps. (b) Extended 2D bands with the non-Abelian topological charges $Q = q_{1234}$. White circles indicate the corresponding 1D bands and red dots represent DPs. (c) The energy spectra of 1D finite tight-binding model with open boundary condition, where red circles represent edge states. (d) The illustration of finite 1D photonic chain with $N = 10$ cells and with perfect electric conductor boundary condition. (e) The energy spectra of 1D photonic chain with $N = 10$. (f) Electric field distributions of bulk states.

When we splice two systems together, then the existence of topological edge states could be characterized by the domain wall charge $\Delta Q = Q_L/Q_R$, where $Q_{R(L)}$ is the non-Abelian topological charge of the left (right) sample. Following this definition, we splice 1D photonic chain with $\theta = 5°$ and $N = 7$ cells with its dual system with $\theta = 175°$ and $N = 7$ cells, as illustrated in Fig. (S7a). Due to that these two systems possess identical non-Abelian topological charge $Q = q_{1234}$, so that the domain wall charge $\Delta Q = 1$, which indicates the domain wall is topological trivial and topological edge state could not exist in the domain wall. In Fig. (S7b), we calculate the energy spectra of the 1D composite photonic chain shown in Fig. (S7a). It shows that in the midgap (marked by orange shadow), there is no edge state. We

then label two bulk states near the midgap as “3” and “4”, and the corresponding electric fields are shown in Fig. (S7d). We can see that the bulk states indeed diffuse nonlocally into the entire 1D composite photonic chain.

At last, we experimentally verify the existence of topological edge states. In Fig. (S7c), we measure the transmission spectra of 1D composite photonic chain. The experimental results (red dashed line) and simulated results (black solid line) fits well with each other. Clearly, there is no edge state in the midgap, which is consistent with the theoretical predictions.

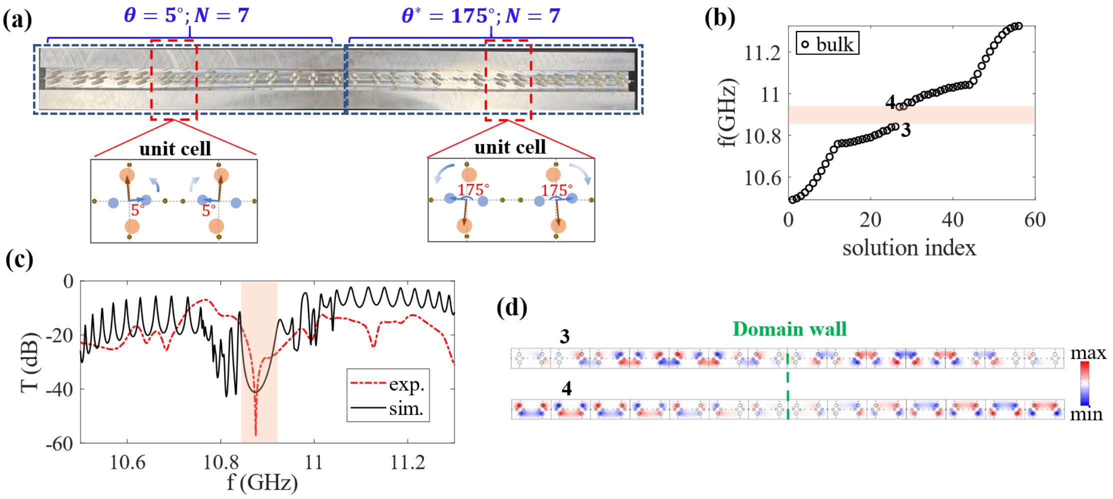

Fig S7. (a) The experimental structure of 1D composite photonic chain with $\theta = 5^\circ$ ($N = 7$ cells) splices with $\theta = 175^\circ$ ($N = 7$ cells). (b) The energy spectra of the 1D composite photonic chain. (c) The transmission spectra of 1D composite photonic chain. The simulated and experimental results are denoted by the black lines and red dotted lines, respectively. (d) Field distributions of bulk states.

Section 6: Non-Abelian topological phase transition in 2D extended bands

In the main text, we have exhibited non-Abelian topological phase transition by Dirac node degeneracy and nodal line degeneracy in 2D extended bands for $\theta = 12.5°$ and $\theta = 90°$, respectively. In this section, we will investigate and visualize the other phase transition process near $\theta = 167.5°$ in the 2D extended bands.

In Fig. (S8a-c), we discuss the non-Abelian topological phase transition process near $\theta = 167.5°$. Hence, we also introduce the radial radius ρ and make substitutions $\cos k \to \rho \cos k = k_1$ and $\sin k \to \rho \sin k = k_2$. Specifically, In Fig. (S8a), we calculate the 2D extended bands for $\theta = 150°$ in $\{k_1, k_2\}$ space, where the white lines represent the original 1D bands. It shows that there are three Dirac nodes inside the unit circle and the topological charge of nodes could be calculated as $Q = q_{12}, -q_{23}$ and q_{34} from bottom band to top band, respectively. Thus, the topological charges of 1D bands could be obtained by the non-Abelian multiplications of nodes inside the unit circle, i.e., $Q = q_{12} \cdot (-q_{23}) \cdot q_{34} = -q_{14}$, which is consistent with the results by orthographic projections of the four eigenstate trajectories, as discussed in the main text. With the increase of θ, a new Dirac point (with topological charge $Q = q_{23}$) out of the unit circle could move towards the boundary of the unit circle. Critically, as shown in Fig. (S8b), it intersects with the unit circle when $\theta = 167.5°$, which is generally regarded as a topological phase transition point [8, 9]. Further increase θ, as shown in Fig. (S8c), the new Dirac point moves in the unit circle and the topological charge of the 1D band could be calculated as $Q = q_{12} \cdot q_{23} \cdot (-q_{23}) \cdot q_{34} = q_{1234}$, which is also in agreement with the results by orthographic projections of the four eigenstate trajectories.

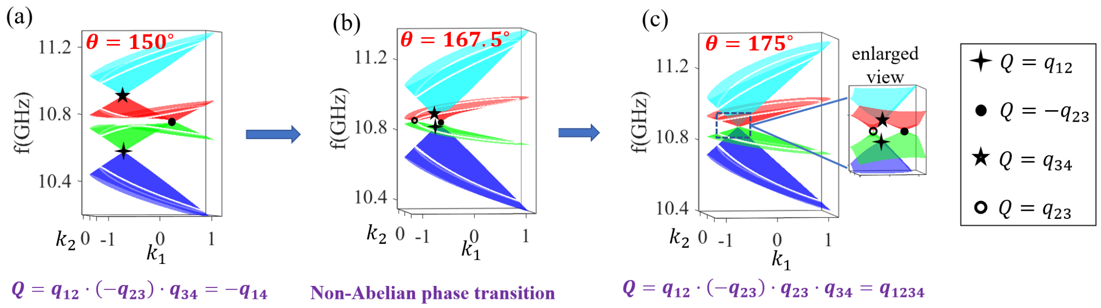

Fig S8. (a)-(c) The 2D extended bands near topological phase transition $\theta = 167.5°$. The topological charges of Dirac points are marked by four-pointed star, pentagram, solid circle, and hollow circle, respectively.

Section 7: A complete set of eigenstate trajectory orthographic projections

In Fig.(3b) in the main text, we have presented orthographic projection of four eigenstate trajectories on to 3D solid sphere in uyz and uzx spaces. The eigenstate trajectory fully characterizes the non-Abelian charge by the rotation directions of four eigenstate trajectories. In this section, we will present the complete set of four eigenstate trajectory projections in all uxy, uxz, uyz and xyz spaces and clarify that these projections are equivalent and any of these four combinations fully characterizes the non-Abelian properties.

In Fig.(S9a) and Fig.(S9b), we calculate orthographic projections of the four eigenstate trajectories onto 3D solid spheres in four spaces for $\theta = 5°$ and $\theta = 175°$, respectively. As these two systems possess identical non-Abelian charge $Q = q_{1234}$, one can find that all four bands flip their signs after k runs across the 1D FBZ with the same rotation direction. Besides, we also depict orthographic projections of the four eigenstate trajectories for $\theta = 30°$ (with $Q = q_{14}$) and $\theta = 150°$ (with $Q = -q_{14}$) in Fig.(S9c) and Fig.(S9d). In all four spaces, only the first band (blue) and fourth band (cyan) flip their signs after k runs across the 1D FBZ, and the rotation direction of eigenstate trajectories is opposite for Fig.(S9c) and Fig.(S9d) due to the reversal non-Abelian topological charge.

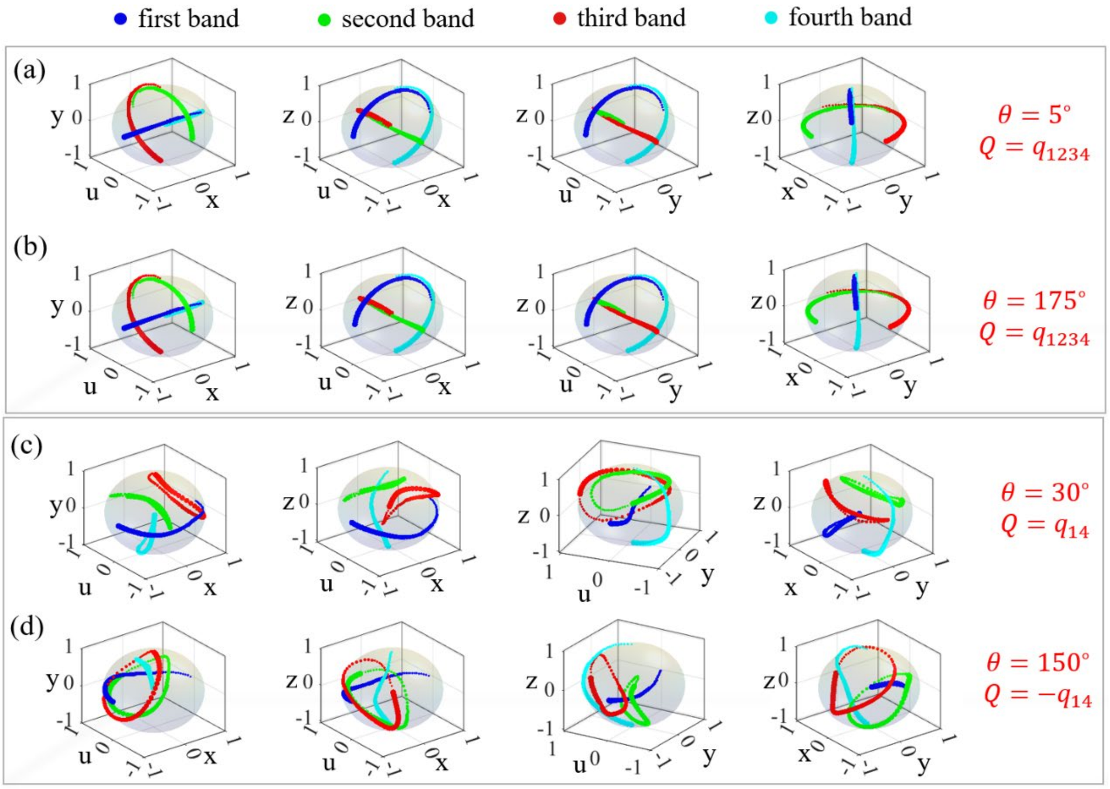

Fig S9. Orthographic projections of the four eigenstate trajectories (shown in different colors) onto 3D solid spheres for (a) $\theta = 5°$, (b) $\theta = 175°$, (c) $\theta = 30°$ and (d) $\theta = 150°$.

Section 8: FDTD simulation for the existence of topological edge states

In the main text, the transmission spectra of Finite Element Method (FEM) simulation clearly support the existence of topological edge state, which is further verified by experimental measurements. In this section, an additional Finite Difference Time Domain (FDTD) simulation is performed to strengthen the validation of the existence of edge states between two systems with different non-Abelian topological phases.

FDTD simulation set up. The FDTD simulation setup is illustrated in Fig. (S10a). The structure consists of PhC-2 ($\theta = 30°, N = 6$), defined in the main text, positioned on the left, and PhC-3 ($\theta = 150°, N = 6$), positioned on the right. The structural and material parameters for each rod are identical to those used in the FEM simulations described in the main text. Perfect electric conductor (PEC) boundary conditions are applied in the y- and z-directions, while a perfectly matched layer (PML) boundary condition is applied in the x-direction. An electric dipole source, indicated by red stars, is placed near the center. Thirty field monitors are then randomly distributed throughout the domain to record the electromagnetic field evolution over time.

Results. By performing Fourier transform on the time-domain field, the amplitude spectra in frequency domain are obtained, as shown in the Fig. (S10b). For comparison, the transmission spectra calculated by FEM simulated (black line) and measured experimentally (red line) are also calculated in Fig. (S10c). It shows that the positions of topological edge states performed by FDTD simulation are in excellent agreement with both the FEM simulation and experimental results. Further, the field distribution of edge states obtained by FDTD simulation, depicted in Fig. (S10d), shows highly localized property at the boundary, which is well consistent with the field distribution obtained from FEM simulation. Thus, the FDTD simulation strongly strengthens the validation of the existence of topological edge states.

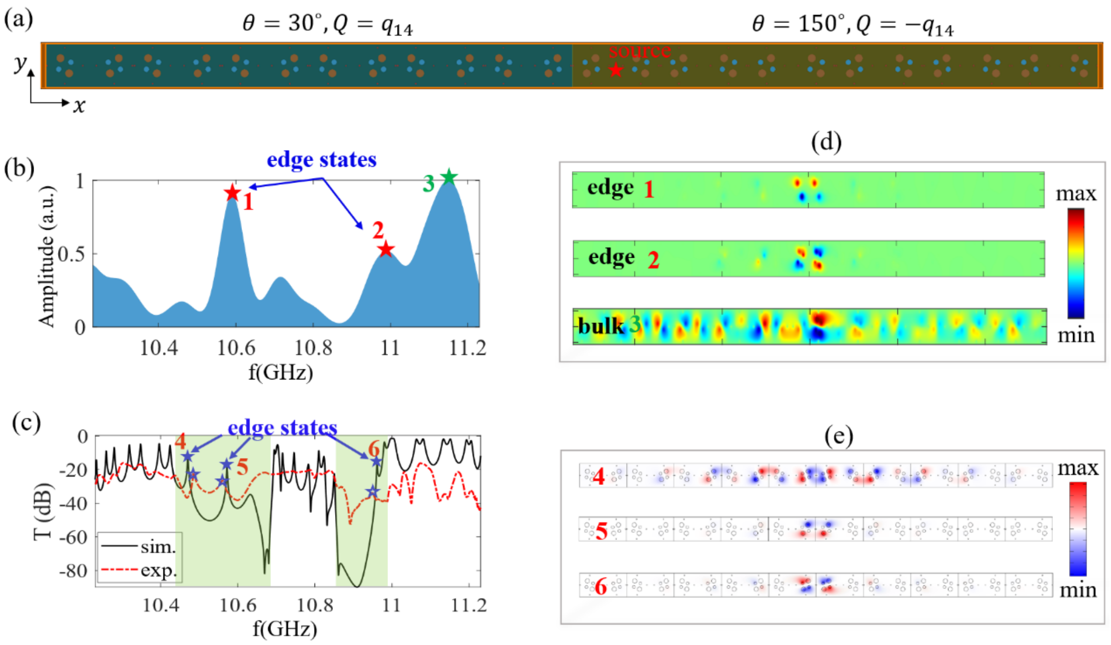

Fig S10. (a) The illustration of FDTD simulation, in which PhC-2 with $\theta = 30°$ ($N = 6$) splices with PhC-3 with $\theta = 150°$ (N = 6). (b) The measured amplitude spectra obtained from FDTD simulation, where edge stats and bulk state are marked by red and green stars. (c) The FEM simulated (black line) and experimental (red line) transmission spectra, in which two bandgaps are marked by green shadows. (d) The field distribution of edge states and bulk state obtained from FDTD simulation. (e) The field distribution of edge states obtained from FEM simulation.

Section 9: The details of experimental setup

The schematic and photograph diagram of experimental setup are shown in Fig. (S11). The ports of PNA-X Network Analyzer N5245B of Keysight are connected to the finite 1D photonic chain with coax-to-waveguide adapters to send and detect the signals. Here, the height of 1D photonic chain is set as 10mm, so that near the operating frequency $f = 11GHz$, only fundamental TE10 could propagate in the 1D photonic chain. Besides, the coax-to-waveguide adapters BJ100 are working between 8.5GHz~12.5GHz and could activate the fundamental TE10 in 1D photonic chain. Finally, the transmission spectra could be obtained from the Network Analyzer.

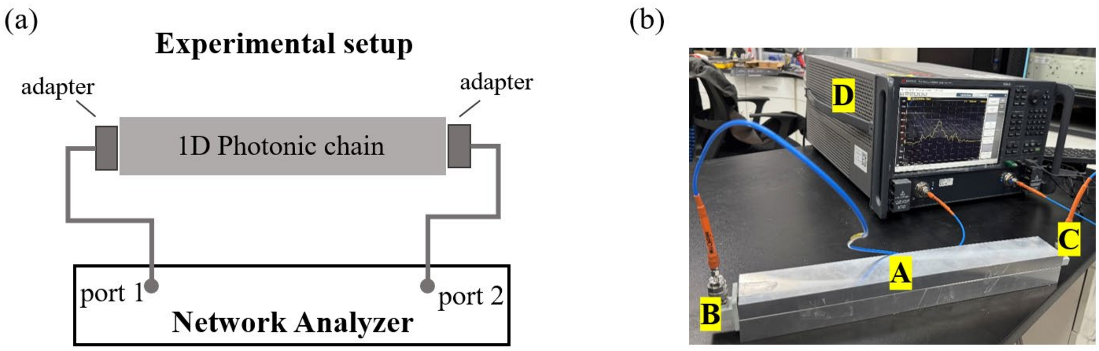

Fig S11. (a) Schematic diagram of experimental setup, in which the coax-to-waveguide adapters operate between 8.5 GHz and 12.5 GHz. (b) Photograph of the experimental setup for the transmission spectra. The 1D Photonic chain (A) is connected to the network analyzer (D) through coax-to-waveguide adapters (B) and (C).

Reference

[1] Gao, Feng, et al. "Orbital topological edge states and phase transitions in one-dimensional acoustic resonator chains." *Nature Communications* 14.1 (2023): 8162.

[2] Lai, Zhen, et al. "The Nested Topological Band-Gap Structure for the Periodic Domain Walls in a Photonic Super-Lattice." *Crystals* 14.9 (2024): 757.

[3] Fruchart M, Yao C, Vitelli V. Systematic generation of Hamiltonian families with dualities[J]. Physical Review Research, 2023, 5(2): 023099.

[4] Fruchart M, Zhou Y, Vitelli V. Dualities and non-Abelian mechanics[J]. Nature, 2020, 577(7792): 636-640.

[5] Benalcazar W A, Bernevig B A, Hughes T L. Electric multipole moments, topological multipole moment pumping, and chiral hinge states in crystalline insulators[J]. Physical Review B, 2017, 96(24): 245115.

[6] He L, Addison Z, Mele E J, et al. Quadrupole topological photonic crystals[J]. Nature communications, 2020, 11(1): 3119.

[7] Slobozhanyuk A P, Poddubny A N, Miroshnichenko A E, et al. Subwavelength topological edge states in optically resonant dielectric structures[J]. Physical review letters, 2015, 114(12): 123901.

[8] Guo Q, Jiang T, Zhang R Y, et al. Experimental observation of non-Abelian topological charges and edge states[J]. Nature, 2021, 594(7862): 195-200.

[9] Jiang T, Guo Q, Zhang R Y, et al. Four-band non-Abelian topological insulator and its experimental realization[J]. Nature communications, 2021, 12(1): 6471.
